# Characteristics of clinical drugs for elderly chronic heart failure complicated with different degrees of renal insufficiency

**DOI:** 10.12669/pjms.341.13600

**Published:** 2018

**Authors:** Hepeng Yu, Jing Chang, Wenwen Zhong, Ping Ye

**Affiliations:** 1 Hepeng Yu, Chinese PLA General Hospital, Beijing 100853, P. R. China; 2 Jing Chang, Chinese PLA General Hospital, Beijing 100853, P. R. China; 3 Wenwen Zhong, Chinese PLA General Hospital, Beijing 100853, P. R. China; 4 Ping Ye, Chinese PLA General Hospital, Beijing 100853, P. R. China

**Keywords:** Elderly, Heart failure, Chronic, Renal insufficiency, Treatment, Drug

## Abstract

**Objective:**

To investigate the characteristics of clinical therapeutic drugs in elderly chronic heart failure (CHF) patients complicated with different degrees of renal insufficiency.

**Methods:**

The elderly patients who were hospitalized from October 2010 to October 2015 in our hospital due to CHF for the first time were selected by means of retrospective case collection. The glomerular filtration rate was estimated by using the Modification of Diet in Renal Disease (MDRD) Study equation. The patients were divided into a group with normal renal function, a group with slight decrease in renal function, and a group with moderate and severe decrease in renal function. Statistical analysis was made to compare the characteristics of clinical drugs for the three groups.

**Results:**

Compared with the normal renal function group and the slight decrease group, ACEIs and β-blockers were less used in the moderate and severe decrease group, but diuretics and spironolactone were more used (P<0.05). Compared with the normal renal function group, the use rate of ACEIs was low whereas that of diuretics was high (P<0.05).

**Conclusion:**

ACEIs and β-blockers were barely employed to treat elderly CHF patients complicated with renal insufficiency, but diuretics and spironolactone were frequently utilized.

## INTRODUCTION

Chronic heart failure (CHF) is a series of complex clinical symptoms caused by the gradual decline of cardiac pump function. It is the terminal stage of most cardiovascular diseases, with a low survival rate, and its 5-year survival rate is even lower than many malignancies. With the gradual acceleration of the current social population aging, as well as the improvement of living conditions, the number of the people suffering with cardiovascular diseases is on the rise, including coronary heart disease, hypertension, diabetes, valvular heart disease, vascular degeneration calcification, atrial fibrillation, dyslipidemia, obesity and atherosclerosis, resulting in the incidence of CHF increased year by year. Some statistics from European and American countries show that about five million US citizens suffer from CHF, with more than 550,000 patients newly diagnosed with CHFs each year, with the incidence for people over 65 years of age reaching 6%-10%.^1^ Renal insufficiency is the most common complications of CHF. ANCHOR, CHARM^2^,^3^ and other large clinical trial results show that the incidence of renal insufficiency in CHF patients is 36%-57%. In this study, the Modification of Diet in Renal Disease (MDRD) Study equation was used to evaluate renal function, and the clinical features of therapeutic drugs for elderly patients with CHF complicated with varying degrees of renal insufficiency were also explored.

## METHODS

This study has been approved by the ethics committee of our hospital, and written consent was obtained from all patients. This study retrospectively selected 436 elderly patients who were hospitalized due to CHF for the first time in our hospital from October 2010 to October 2015. All the patients were in line with Framingham heart failure diagnostic criteria.^4^ The selected cases were aged 60-98 years, with the median age of 79 years, including 322 males (73.9%) and 114 females (26.1%). Cardiac function was classified into grade II-IV by cardiac function grading standard (NYHA classification of cardiovascular disease) of New York Cardiology Association, of which there were 175 cases of cardiac function grade II (40.1%), 141 cases of grade III (32.3%) and 120 cases of grade IV (27.5%). Among the selected cases, the etiology of CHF included 255 cases of coronary heart disease (58.5%), 72 cases of high heart disease (16.5%), 16 cases of rheumatic heart disease (3.7%), 38 cases of dilated cardiomyopathy (8.7%), 29 cases of pulmonary heart disease (6.7%), and 26 cases of other causes (6.0%).

The glomerular filtration rate (eGFR) was estimated according to the serum creatinine level at admission, and calculated using the simplified MDRD Study equation:^5^ eGFR ml/(min•1.73 m^2^) = 186 × [creatinine] - 1.154 × [age] - 0.203 × 1.233 (female × 0.742). The unit of creatinine was used as mg/dl in the equation (1 mg/dl = 88.402 μmol/L). According to the results of eGFR, 436 patients were divided into three groups: the group with normal renal function: eGFR ≥90 ml/(min•1.73 m^2^), i.e. CKD phase I, 176 cases in total; the group with slight decrease in renal function: eGFR 60-89 ml/(min•1.73 m^2^), i.e. CKD phase II, 141 cases in total; the group with moderate and severe decrease in renal function: eGFR <60 ml/(min•1.73 m^2^), i.e. CKD phase III-V, 119 cases in total. Statistical analysis was performed on the general situation at admission and the characteristics of clinical drugs in elderly CHF patients with different degrees of renal insufficiency.

### Statistical analysis

SPSS v17.0 statistical software was used for statistical analysis. The categorical data in line with normal distribution were expressed as (mean ± standard deviation), and those not in line with normal distribution were expressed as median and 25% and 75% percentiles. For the data of normal distribution, three groups were compared by using one-way analysis of variance, and pairwise comparisons were carried out with the q test. The data not conforming to normal distribution were expressed as median (P25, P75), using the rank sum test.

## RESULTS

### Baseline clinical data

Compared with the normal renal function group and the slight decrease group, the moderate and severe decrease group was older, with fewer cases of grade II cardiac function and more cases of grade IV (P<0.05). Compared with the normal renal function group, the slight decrease group was older and had more cases of grade IV cardiac function (P<0.05) (Table-I).

**Table-I:** Baseline clinical data [case number (%)].

Item	Total case number (436)	Normal renal function group (176)	Slight decrease group (141)	Moderate and severe decrease group (119)
Gender (male/female) (case)	322/114	118/58	100/41	104/15
Age (year)	79 (60, 98)	67 (63, 85)	78 (65, 89)^a^	81 (71, 98)^bc^
*Cardiac function classification (grade)*				
II	175 (40.1)	93 (52.8)	49 (34.8)	33 (27.7)^bc^
III	141 (32.3)	62 (35.2)	43 (30.5)	36 (30.3)
IV	120 (27.5)	21 (11.9)	49 (34.8)^b^	50 (42.0)^bd^

Compared with normal renal function group,

a P<0.05,

b P<0.01;

Compared with slight decrease group,

c P<0.05,

d P<0.01.

### Treatment outcomes of clinical drugs

Compared with the normal renal function group and the slight decrease group, ACEIs and β-blockers were less used in the moderate and severe decrease group, but diuretics and spironolactone were more used (P<0.05). Compared with the normal renal function group, the use rate of ACEIs was low whereas that of diuretics was high (P<0.05). The three groups used similar proportions of nitric esters, ARBs, digoxin, aspirin, clopidogrel and statins (P>0.05) (Table-II).

**Table-II:** Treatment outcomes of clinical drugs.

Drug	Normal renal function group (176)	Slight decrease group (141 cases)	Moderate and severe decrease group (119 cases)
Nitric esters, n (%)	101 (57.4%)	75 (53.2%)	45 (37.8%)
ACEIs, n (%)	105 (59.7%)	71 (50.4%)^a^	51 (42.9%)^bc^
ARBs, n (%)	46 (26.1%)	30 (21.3%)	29 (24.4%)
β-Blockers, n (%)	83 (47.2%)	65 (46.1%)	44 (37.0%)^bc^
Diuretics, n (%)	97 (55.1%)	82 (58.2%)^b^	89 (74.8%)^bc^
Spironolactone, n (%)	71 (40.3%)	69 (48.9%)	68 (57.1%)^c^
Digoxin, n (%)	16 (9.1%)	12 (8.5%)	17 (14.3%)
Aspirin, n (%)	122 (69.3%)	85 (60.3%)	73 (61.3%)
Clopidogrel, n (%)	91 (51.7%)	67 (47.5%)	49 (41.2%)
Statins, n (%)	73 (41.5%)	73 (51.8%)	50 (42.0%)

Compared with normal renal function group,

a P<0.05,

b P<0.01;

Compared with slight decrease group,

c P<0.05,

d P<0.01.

## DISCUSSION

Up to now, there has been a lack of large-scale clinical trial data in the treatment of CHF patients with renal insufficiency, and whether the drugs for the treatment of CHF are beneficial to them has also not been well confirmed yet. However, most of the current methods are empirical treatment. Poor CHF control will lead to a rapid decline of creatinine clearance rate at one mL/minute per month, and renal dysfunction will further aggravate the progress of heart failure, causing a vicious cycle. Therefore, it is of great significance for treatment combined with active correction of heart failure and renal failure to improve the prognosis of CHF patients with renal insufficiency.

Rennin-Angiotensin-Aldosterone system (RAAS) antagonists are drugs that can block the role of RAAS, including ACEIs and ARBs. For the heart, RAAS antagonists can delay myocardial remodeling, improve cardiac function, reduce the risk of death, and improve the survival rate of CHF patients. For the kidneys, they can also selectively expand efferent arteriole, thereby reducing intraglomerular pressure and proteinuria, inhibiting renal tissue sclerosis, and delaying the deterioration of renal function, which are the most effective drugs for kidney protection.^6^ ACEI is the cornerstone medication of CHF therapy. Several large-scale multicenter clinical trials have demonstrated the benefits of ACEI in the treatment of CHF, making it the first choice for ACC/AHA to be used to treat CHF. In addition, during the treatment of CHF, ACEI can also delay the progress of chronic kidney disease. ACEI can reduce the mortality of CHF patients with renal insufficiency and the incidence of cardiovascular events. In recent years, clinical observation data for ARB treatment of CHF has been significantly increased. In particular, in CHARM additional test, 2,028 heart failure patients who had ACEI intolerance received candesartan treatment, of which the mortality of main-endpoint cardiovascular disease or the hospitalization rate of deterioration of heart failure was decreased by 23%. This also helps improve the status of ARB drugs in the treatment of heart failure.

In this study, the utilization rates of 436 patients receiving ACEI were 52.1% and ARB 24.1%, respectively, the latter of which is relatively low. The results still lag behind the requirements in foreign heart failure treatment guidelines. The reasons may lie in the over-concerns of physicians about the adverse reactions of ACEI and ARB drugs, and lack of full attention to their therapeutic effects in heart and kidney protection. CONSENSUS study was conducted on patients with severe heart failure, including those with renal impairment, whose creatine deficiency was less than 3.4 g/L.^7^ In the severe heart failure patients receiving ACEI, over 30% had increased creatinine level, but only a few needed to stop treatment. Moreover, after stopping ACEI treatment, creatinine of the majority of the patients can be restored to baseline levels. The use of ACEI in patients with critical renal insufficiency can still gain long-term benefits. It is required to use routinely unless the patients have routine contraindications. According to the domestic ESBARL study of Hou et al.,^8^ patients with serum creatinine levels of 3-5 g/L, took 20 mg of lotensin daily under close observation, and were observed for 3.4 years on average; and compared with the control group, the risk of advanced chronic kidney disease progressing to end-stage renal failure was reduced by 43%. However, in the course of treatment, ACEI must be started from a small dose, which is gradually increased, and close monitoring is conducted on the patient's serum potassium and creatinine levels, which is often combined with diuretics. In CHF patients who have already started using ACEI, if renal insufficiency occurs, ACEI cannot be disused at will. Therefore, it is believed in the current ACEI treatment that taking into account its role in the protection of heart and kidney, such drugs should be actively used in clinical work as long as there is no progressive deterioration of renal function and the occurrence of severe hyperkalemia, in order to give full play to its role of protection.

The use of diuretics is still a controversial issue in the treatment of CHF with renal insufficiency. Gottlieb et al.^9^ studied the effect of diuretics on renal function, and found that in patients with congestive heart failure who used a single diuretic, the glomerular filtration rate was significantly reduced despite of the increase in urine volume. Nohria et al. is of the view that the use of diuretics in patients with severe CHF may lead to activation of neurohormones, resulting in decreased heart and kidney function, so as to cause diuretic resistance and increased patient mortality. Weinfeld et al.^10^ found that large doses of diuretics can cause deterioration of renal function, especially combined with ACEI drugs. Hence, it is a great challenge for the treatment of CHF patients with renal insufficiency, excessive capacity overload and insensitive to diuretics. Whether the rational use of diuretics can achieve the success of CHF treatment is one of the key factors.

### Authors' contributions

***HY & PY:*** Study design and manuscript preparation.

***JC & WZ:*** Clinical data collection and analysis.
